# Enteritis of the ileal conduit as an adverse event related to immune checkpoint inhibitor use

**DOI:** 10.1002/iju5.12797

**Published:** 2024-10-14

**Authors:** Ko Kobayashi, Kohei Hashimoto, Toshiaki Tanaka, Naoya Masumori

**Affiliations:** ^1^ Department of Urology Sapporo Medical University School of Medicine Sapporo Japan

**Keywords:** bladder cancer, immune‐related adverse events, pembrolizumab, urothelial carcinoma

## Abstract

**Introduction:**

Gastroenterocolitis is one of the adverse events related to immune checkpoint inhibitors. However, inflammation of the intestinal lesion used for urinary diversion is not well known as an adverse event related to their use.

**Case presentation:**

A patient with metastatic bladder cancer was administered pembrolizumab as second‐line treatment. After 12 days of administration, he felt abdominal distention. Computed tomography demonstrated thickening of the ileal conduit wall and bilateral hydronephrosis. Biopsy of the ileal conduit revealed inflammatory granulation tissue. Biopsy of the sigmoid colon also revealed colitis. Therefore, we diagnosed enterocolitis including the ileal conduit related to pembrolizumab. We then started to administer 30 mg prednisolone. After this treatment, we confirmed improvement of the clinical symptoms and healing of the ileal conduit mucosa.

**Conclusion:**

Inflammation of an ileal conduit can occur as an immune‐related adverse event caused by metastatic urothelial carcinoma treatment with pembrolizumab.

**Keynote message**

We report a case of inflammation of the ileal conduit as an immune‐related adverse event with pembrolizumab. The use of an immune checkpoint inhibitor has the possibility of causing inflammation of the intestine used for urinary diversion as an immune‐related adverse event.

**Abbreviations & Acronyms**
CT: computed tomography
CTLA‐4: cytotoxic T‐lymphocyte antigen 4
ICI: immune checkpoint inhibitor
irAEs: immune‐related adverse events
NCCN: National Comprehensive Cancer Network
PD‐1: programmed death 1
PD‐L1: programmed death ligand 1

## Introduction

Several studies have revealed the benefits of immunotherapy using ICI for treatment of metastatic urothelial carcinoma.^1^ The NCCN Guidelines introduce four ICIs (pembrolizumab, nivolumab, atezolizumab, and avelumab) for systemic therapy in metastatic bladder cancer.^2^ Additionally, it has been suggested that adjuvant nivolumab for patients with high‐risk muscle invasive urothelial carcinoma who have undergone radical surgery results in longer disease‐free survival than a placebo.^3^ Thus, it seems likely that more and more patients with advanced urothelial carcinoma will receive immunotherapy using ICI. On the other hand, physicians have to be aware of specific adverse events caused by ICI called irAEs. A systematic review reported that the incidence of any grade irAEs was 34.3% and that of grade ≧3 was 10.2% in patients with urologic cancer.^4^ Gastrointestinal adverse event is one of the irAEs, and the incidence of any grade colitis as an irAE is 2.9% in urologic cancer patients.^4^ Gastritis and enteritis were also reported as gastrointestinal irAEs when using ICI.^5^ However, inflammation of the intestinal segment used in urinary diversion is not well known as an irAE. We report a case of inflammation of the ileal conduit related to metastatic urothelial carcinoma treatment with pembrolizumab.

## Case presentation

**Fig. 1:**
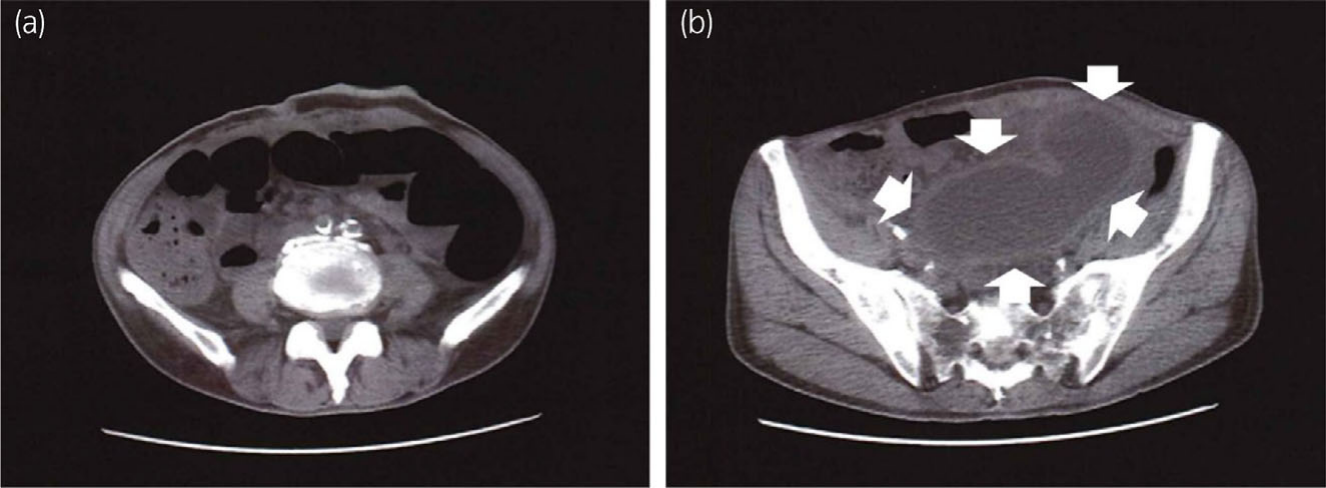
CT shows (a) retention of colon gas and (b) extension and wall thickness (white arrow) of the ileal conduit.

**Fig. 2:**
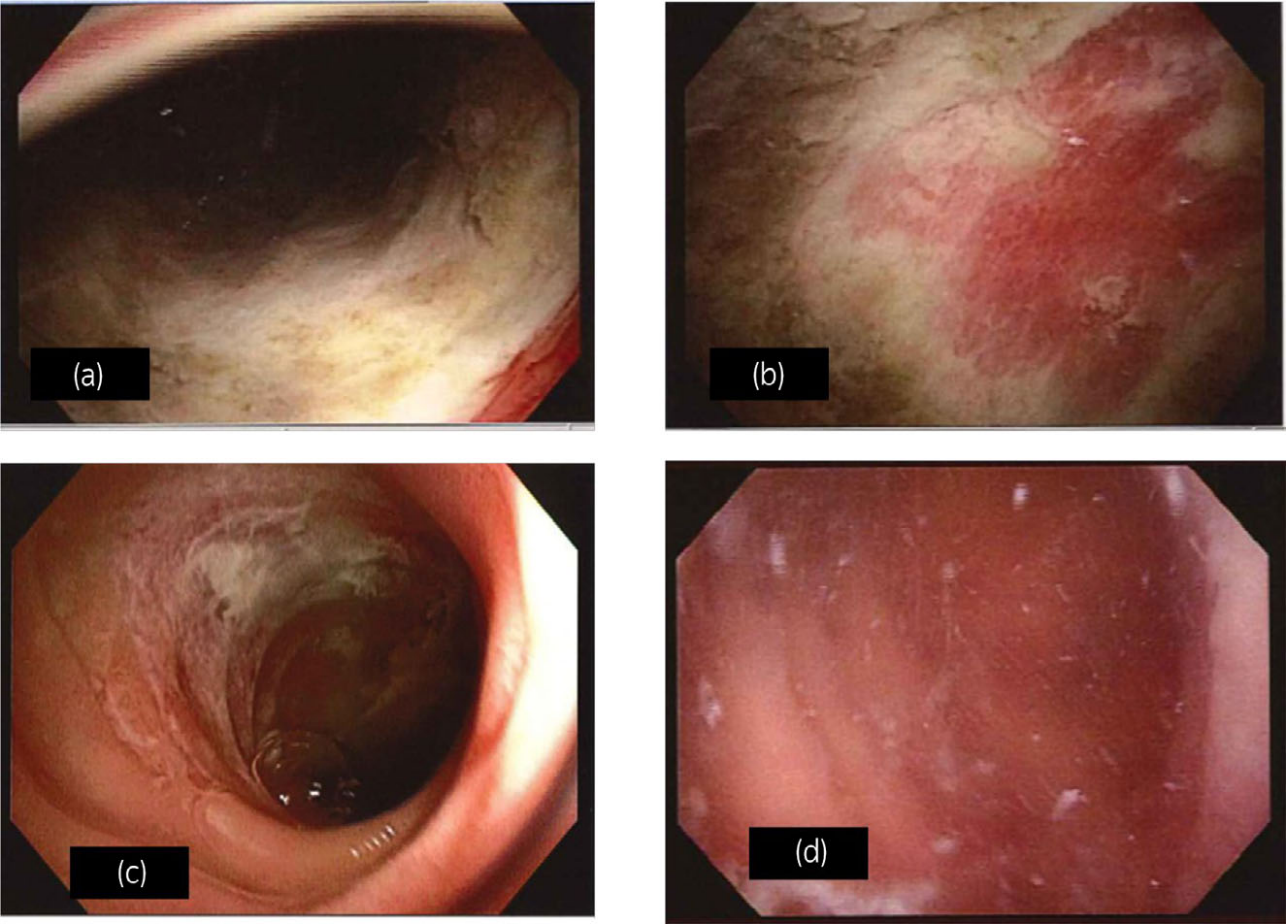
Endoscopic findings of the ileal conduit. The first examination showed that the ileal conduit mucosa was diffusely white and ischemic (a and b). Five days after starting 30 mg prednisolone, endoscopy of the ileal conduit shows mucosal healing (c). Twenty‐five days after beginning the treatment, endoscopic findings of the ileal conduit were much improved (d).

A 69‐year‐old man received laparoscopic radical cystectomy and construction of an ileal conduit for cT4N0M0 bladder cancer. Pathological findings revealed pT3aN3 urothelial carcinoma. After radical surgery, he received three courses of adjuvant chemotherapy (gemcitabine and cisplatin: GC). Then, he was followed by radiological examination. After 3 years, follow‐up CT showed para‐aortic lymph node swelling. We diagnosed it as lymph node metastasis and started chemotherapy (GC) for him. After two courses of GC treatment, multiple bone metastasis newly occurred. We decided to start immunotherapy using pembrolizumab as second‐line therapy for metastatic bladder cancer. We planned intravenous pembrolizumab 200 mg every 3 weeks. At 13 days after the first pembrolizumab administration, he visited our hospital because of abdominal distension. CT revealed retention of colon gas, extension, and wall thickness of the ileal conduit and bilateral hydronephrosis (Fig. 1). His blood test showed creatinine and potassium levels elevated to 3.89 mg/dL and 6.6 mEq/L, respectively. Based on CT findings, we suspected his condition to be ileus and urinary retention in the ileal conduit. He was hospitalized and treated with intravenous fluids and drainage of urine from the ileal conduit via a temporary indwelling soft catheter. However, his hydronephrosis still remained after the indwelling the catheter. The day after admission, we performed colonoscopy. Biopsy of the sigmoid colon also revealed colitis. We also performed contrast radiography of the ileal conduit and found decreased peristalsis and urinary excretion of the ileal conduit. Then, we performed endoscopy of the ileal conduit. This examination showed that the ileal conduit mucosa was diffusely white and ischemic (Fig. 2a,b).

**Fig. 3:**
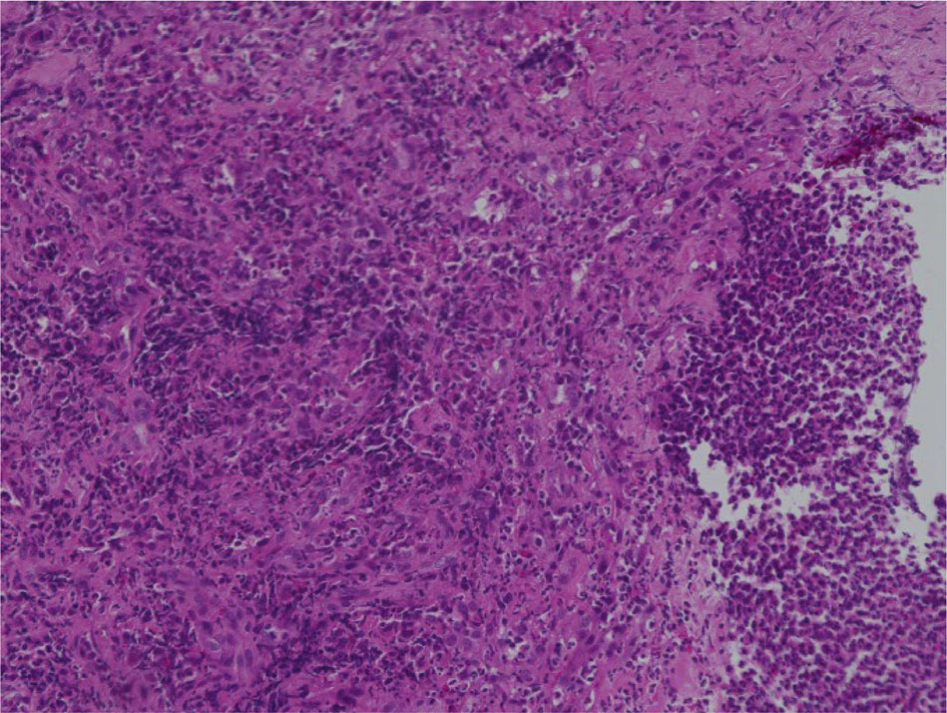
Histological examination revealed inflammatory granulation tissue with severe neutrophil infiltration (objective ×20).

Biopsy of the ischemic lesion from the ileal conduit revealed inflammatory granulation tissue (Fig. 3). The biopsy from the white lesion showed only debris. Therefore, we diagnosed enterocolitis including the ileal conduit related to pembrolizumab. We then started to administer 30 mg prednisolone. Five days after starting this treatment, we confirmed mucosal healing of the ileal conduit (Fig. 2c) and improvement of the clinical symptoms including hydronephrosis. A blood test showed that creatinine and potassium levels improved to 1.20 mg/dL and 4.7 mEq/L, respectively. Twenty‐five days after the prednisolone treatment, endoscopic findings of the ileal conduit were much improved (Fig. 2d). We gradually tapered the prednisolone dose to 15 mg without any worsening of symptoms and removed the catheter from the ileal conduit. However, the patient's overall condition deteriorated due to progression of the disease. Thereafter, he was transferred to a palliative care hospital and died of cancer 2 months later.

## Discussion

Gastrointestinal toxicity due to ICI is relatively common, and diarrhea has been reported in up to half of patients, with the reported incidence typically between 30% and 40%.^6^ Other gastrointestinal irAEs include nausea, abdominal pain, constipation, gastroesophageal reflux disease, hemorrhagic enteritis, intestinal obstruction, necrotizing colitis, and gastrointestinal perforation.^7^ In addition, other reports have suggested that PD‐1/PD‐L1 antibodies (and other similar drugs, including CTLA‐4 antibodies) can cause gastritis, enteritis, and colitis, and the histological findings vary depending on the drug.^5^ In this context, gastrointestinal irAEs can occur in any part of the intestinal tract. In this case, based on the findings and treatment course, it was thought that irAE‐related inflammation had also developed in the intestinal tract of the ileal conduit.

Adjuvant nivolumab therapy is one of the recommended regimens for patients received radical cystectomy and urinary diversion, who are considered at high risk for disease recurrence.^2^, ^3^, ^8^ Systemic therapy with pembrolizumab or avelumab is indicated for locally advanced or metastatic bladder cancer in Japan.^8^ Patients with metastatic disease include those with urinary diversion. Therefore, the chance of using ICI for these patients will continue to increase. We have to be aware of various possible adverse events during ICI usage. Considering this case, we should recognize the possibility of irAEs also occurring in the intestine used for urinary diversion.

There are two main clinical phenotypes of small bowel involvement‐related ICIs. One is characterized by enteropathy with villous atrophy, whether or not related to celiac disease, and the other is characterized by generally severe ulcerative enteritis, sometimes with massive gastrointestinal bleeding or perforation of the small intestine but without any villous atrophy.^9^ In general, the enteropathy with villous atrophy type occurs in patients with ICI monotherapy.^9^ Our case might have had this type of inflammation because we could not observe villous architecture in the lesion that we biopsied and the patient received pembrolizumab monotherapy.

Systemic steroid therapy is recommended for moderate to severe adverse gastrointestinal events.^6^, ^7^ In this case, inflammation of the ileal conduit improved rapidly with the administration of prednisolone. To the best of our knowledge, this is the first case report of an irAE occurring the intestine used for urinary diversion. Therefore, no definitive treatment for this condition has been established. However, we believe that treatment of irAEs occurring in the intestine used for urinary diversion may be similar to that for general immune‐related gastrointestinal disorders.

## Conclusions

We reported a case of inflammation of an ileal conduit caused by treatment with pembrolizumab. Doctors should thus be aware of the possibility that ICI treatment can cause irAE inflammation of the ileal segment used for urinary diversion.

## Author contributions

Ko Kobayashi: Conceptualization; data curation; methodology; project administration; writing – original draft; writing – review and editing. Kohei Hashimoto: Conceptualization; data curation; project administration. Toshiaki Tanaka: Conceptualization; data curation; project administration; supervision. Naoya Masumori: Supervision.

## Conflict of interest

The authors declare no conflict of interest.

## Approval of the research protocol by an Institutional Reviewer Board

N/A.

## Informed Consent

N/A (the patient is dead).

## Registry and the Registration No. of the study/trial

N/A.
